# Antimicrobial Resistance Patterns of Clinically Isolated Strains of Acinetobacter baumannii in a Tertiary Care Hospital in Libya

**DOI:** 10.7759/cureus.76483

**Published:** 2024-12-27

**Authors:** Dalal Thwood, Zynab Alghadem, Nada Elgriw, Maren Hnaya, Ahmed Zaghdani, Salah Edin El Meshri, Adem Elzagheid

**Affiliations:** 1 Department of Microbiology, Libyan Biotechnology Research Center, Tripoli, LBY; 2 Department of Microbiology, Tripoli University Hospital, Tripoli, LBY; 3 Department of Genetic Engineering, Libyan Biotechnology Research Center, Tripoli, LBY

**Keywords:** acinetobacter baumannii, antibiogram, antimicrobial resistance, clinical specimens, mdrab

## Abstract

Objective: *Acinetobacter baumannii* accounts for a notable percentage of hospital-acquired infections. The widespread resistance to multiple antibiotic classes complicates treatment efforts. This study aims to find out the pattern of susceptibility of multidrug-resistant *A. baumannii* (MDRAB) isolated from clinical specimens to antibiotics recommended for testing and use for* A. baumannii *and to determine a local guide at Tripoli University Hospital (TUH), Tripoli, Libya for the empirical antibiotic treatment of MDRAB based on the susceptibility pattern identified.

Materials and methods: This retrospective cross-sectional study was carried out in the Department of Microbiology at TUH in Libya from January 2021 to June 2021. Identification of isolates was performed using the VITEK® 2 automated system (bioMérieux, France), and the isolates were categorized as susceptible, intermediate susceptible, or resistant according to the minimum inhibitory concentration (MIC).

Results: A total of 86 *A.* *baumannii* isolates were obtained from various clinical specimens from different departments. Isolates show high resistance to most of the tested antibiotics. Beta-lactams and ciprofloxacin were found to be the most resistant, while the highest susceptibility was detected to minocycline and amikacin.

Conclusion: These findings prompt clinicians to consider taking minocycline and amikacin into account when starting empirical therapy for severe infections caused by *A.* *baumannii*.

## Introduction

Antimicrobial resistance (AMR) is a significant and growing problem in the healthcare systems worldwide. It has been linked to increased morbidity, mortality, and cost [1]. *Acinetobacter baumannii*, a gram-negative coccobacillus, predominantly causes nosocomial infections in debilitated, hospitalized patients and prolonged antibiotic use [2,3]. These infections are diverse, including ventilator-associated pneumonia, urinary tract infections, meningitis, bacteremia, and gastrointestinal and skin/wound infections [4]. Intensive care units (ICUs) are considered the main source of *A. baumannii* spread to general wards and long-term healthcare facilities [1]. *A. baumannii* has developed multidrug resistance through various mechanisms, for example, decreased expression of outer membrane porins, the expression of efflux pumps, production of certain β-lactamases, the presence of a "resistance island" comprising multiple resistance genes, and horizontal acquisition of foreign resistance determinants [5].

Multidrug-resistant *A. baumannii* (MDRAB) shows resistance to more than one agent in three categories of antibiotics, making it extremely challenging to treat and control [5,6]. MDRAB is commonly associated with catheter use, mechanical ventilation, and extended hospital stays in immunocompromised patients [7]. Nevertheless, the increased utilization of third-generation cephalosporins has been linked to an increased incidence of nosocomial-acquired pneumonia due to MDRAB. Carbapenems were considered the preferred treatment for MDRAB infection. However, their use has led to an increase in the incidence of carbapenem-resistant isolates in recent years [4]. Despite their nephrotoxicity and neurotoxicity, polymyxins are now widely used as the antibiotics of choice for MDRAB infections, usually in combination with other drugs [8]. WHO has included *A. baumannii* in its list of priority pathogens as a priority one (critical) pathogen to guide research, discovery, and development of new antibiotics [9]. Continuous surveillance of AMR in *A. baumannii* is crucial for selecting appropriate empirical therapies to increase the chances of patient survival [10]. In Libya, there are no local guidelines based on antibiograms or surveillance programs for AMR, and the information about resistance patterns that guide the empirical therapy is limited; therefore, we conducted this preliminary study at Tripoli University Hospital (TUH), Tripoli, Libya, that aims to find out the pattern of susceptibility of MDRAB isolated from clinical specimens to antibiotics recommended for testing and use for* A. baumannii* according to the Clinical Laboratory Standards Institute (CLSI) [11] and to determine a local guide at TUH for the empirical antibiotic treatment of MDRAB based on the susceptibility pattern identified.

## Materials and methods

Setting

An institutional-based cross-sectional study was conducted through a retrospective examination of culture results from the diagnostic microbiology department in the central laboratory of TUH during the period from January 2021 to June 2021. TUH is a multispecialty tertiary care facility, accommodating over 1000 beds, situated in the capital of Libya. The data was collected after the permission of the hospital administration and the laboratory manager to review the results of the microbial cultures while maintaining the confidentiality of the patients' information.

We analyzed 86 non-repetitive strains of MDRAB isolated from various hospital departments, including ICUs, neonatal units, surgical wards, pediatric oncology units, as well as medical and outpatient services. They were isolated from the following clinical specimens: endotracheal tube (ETT), bronchoalveolar lavage (BAL), sputum, suction tube tips, swabs, central venous catheter (CVC), blood cultures, and cerebrospinal fluid (CSF).

We review the antimicrobial susceptibility result of *A. baumannii *according to the Clinical and Laboratory Standards Institute (CLSI, 2022) [11], which divided the antibiotics tested for *A. baumannii *into two groups: Group A antibiotics that were considered appropriate for inclusion in the routine primary testing panel and routine reporting of the results and group B antibiotics that may warrant primary testing but may be reported only selectively. Group A includes the following antibiotics: ampicillin-sulbactam (AMS), ceftazidime (CAZ), imipenem (IMI), meropenem (MEM), doripenem, gentamicin (CN), tobramycin (TOB), ciprofloxacin (CIP), and levofloxacin (LEV). Group B included piperacillin/tazobactam (PTZ), ceftriaxone (CRO), cefepime (FEP), amikacin (AK), trimethoprim/sulfamethoxazole (TMP/SMZ), minocycline (MIN), cefiderocol, and doxycycline.

The identification of *A. baumannii* and antimicrobial susceptibility testing identification was conducted utilizing the VITEK® 2 automated system (bioMérieux, France). Following the isolation of pure colonies on blood and MacConkey agar plates after an overnight incubation period, gram-negative identification and antimicrobial susceptibility testing cards (AST-GN 75, 22) were employed to ascertain the antibiotic susceptibility of *A. baumannii*. Both testing cards were inoculated with a bacterial suspension that matched the turbidity of a 0.5 McFarland standard. The panel included the following antibiotics: AMS (10/10 µg), PTZ (100/10), CAZ (30 µg), CRO (30 µg), FEP (30 µg), MEM (10 µg), IMI (10 µg), CN (10 µg), TOB (10 µg), AK (30 µg), CIP (5 µg), MIN (30 µg), colistin (CT) (10 µg), LEV (5 µg), and TMP/SMZ (1.25/23.75 µg). The doripenem, cefiderocol, and doxycycline have not been tested (the available cards do not contain these antibiotics). While colistin was tested, though, it is not recommended to be used as an automated system to test the MIC, as the result is not reliable according to CLSI (2022) [11].

Data analysis

Data were systematically entered and subjected to analysis utilizing IBM SPSS Statistics for Windows, Version 20 (Released 2011; IBM Corp., Armonk, New York, United States), employing simple frequency assessments to illustrate the distribution of *A. baumannii* across various departments and clinical specimens. The antimicrobial resistance pattern of *A. baumannii* was reviewed and compared with that reported in previous studies.

## Results

Of the 86 MDRAB, 42 (48.8%) were from ICUs and 26 (30.2%) from the neonatal unit. Isolation from the pediatric intensive care unit (PICU) (22.1%) was the most common among all ICUs (Table 1).

**Table 1:** Distribution of MDRAB among different departments MDRAB: Multidrug-resistant *Acinetobacter baumannii*

Department	Frequency	Percentage
Intensive care units	42	48.8
Pediatric intensive care unit	19	22.1
Surgical intensive care unit	11	12.8
Medical intensive care unit	7	8.1
Cardiology intensive care unit	5	5.8
Neonatal unit	26	30.2
Surgical ward	10	11.6
Pediatric oncology	4	4.7
Medical ward	3	3.5
Outpatient department	1	1.2
Total	86	100

More than half of *A. baumannii* was isolated from respiratory specimens (48, 55.8%). The highest percentage among them was from ETT (36, 41.9%). Swabs were the second source (19, 22.1%). Seven (8.1%) were isolated from blood cultures as demonstrated in Table 2.

**Table 2:** Distribution of MDRAB according to site of infection MDRAB: Multidrug-resistant* Acinetobacter baumannii*

Specimen	Frequency	Percentage
Respiratory specimens	48	55.8
Endotracheal tube tip	36	41.9
Bronchoalveolar lavage	5	5.8
Sputum	5	5.8
Suction tube tip	2	2.3
Swabs	19	22.1
Wound swabs	10	11.6
Other swabs	9	10.5
Central venous catheter	8	9.3
Blood culture	7	8.1
Urine	3	3.5
Cerebrospinal fluid	1	1.2
Total	86	100

**Figure 1:**
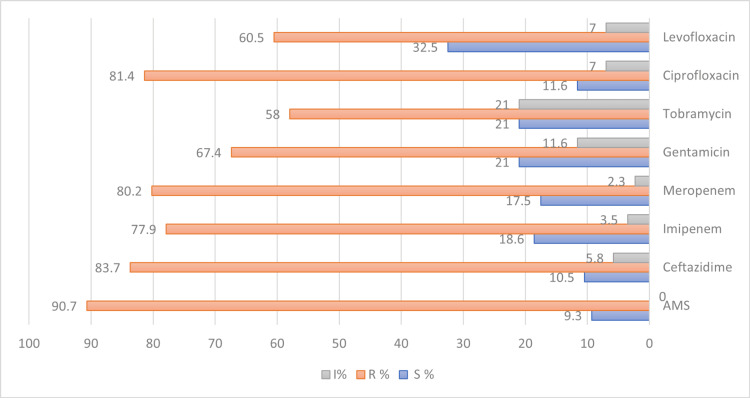
Antimicrobial susceptibility profile of the Acinetobacter baumannii for agents considered appropriate for inclusion in a routine primary testing panel and routine reporting of the results according to CLSI (2022) AMS: ampicillin/sulbactam; S: susceptible; R: resistant; I: intermediate; CLSI: Clinical Laboratory Standards Institute

A high percentage (>50%) of isolates was resistant to most of the antibiotics tested. The highest resistance rates were observed for AMS (90.7%), CAZ (83.7%), CIP (81.4%), and MEM (80.2%). In contrast, the lowest resistance rate was recorded for TOB (58%) and LEV (60.5%) among group A antimicrobial agents. In addition, aminoglycosides showed the highest intermediate levels of resistance as presented in Figure 1.

**Figure 2:**
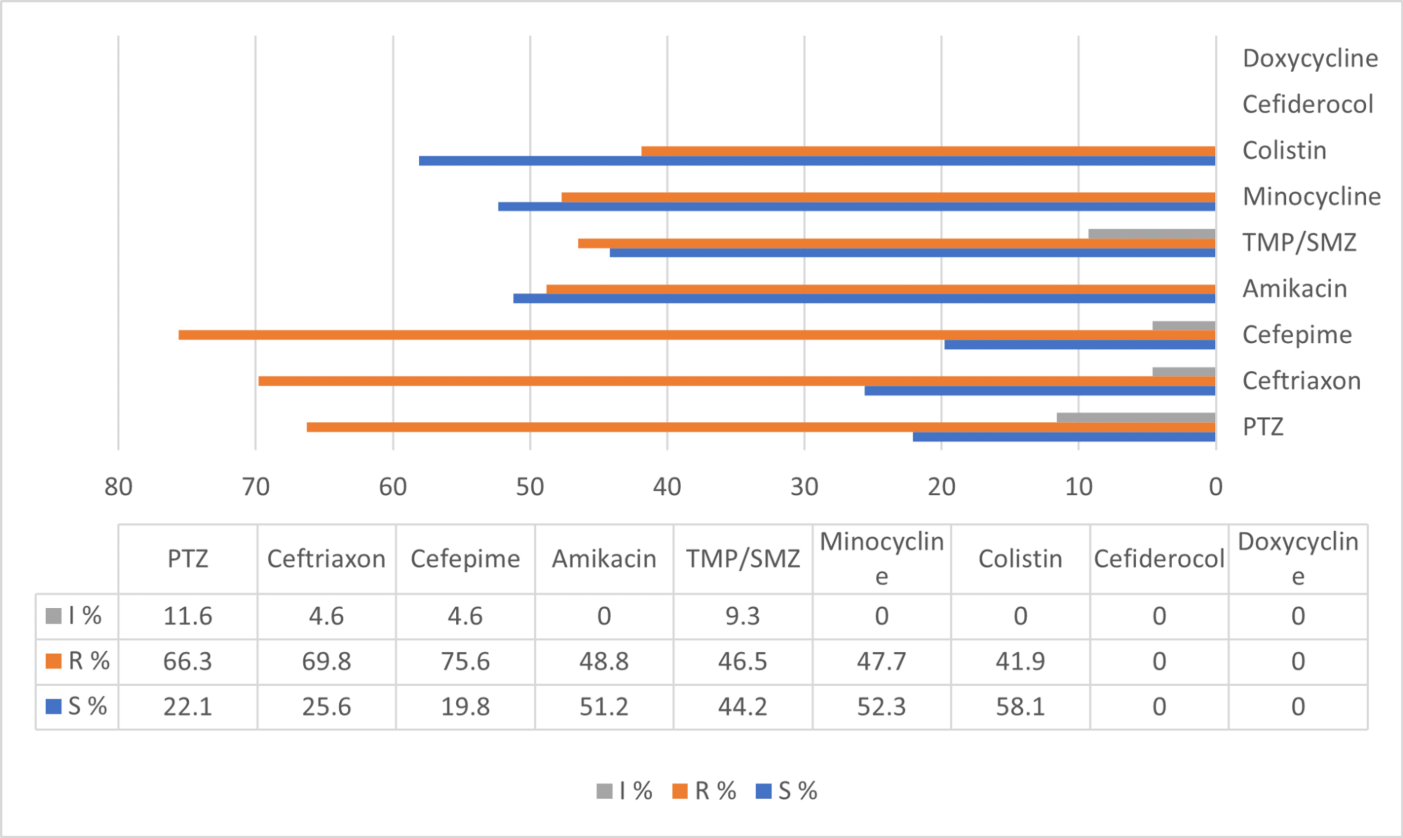
Antibiogram of the antimicrobial agents that may warrant primary testing but may be reported only selectively (group B) for Acinetobacter baumannii according to CLSI (2022) PTZ: piperacillin/tazobactam; S: susceptible; R: resistant; I: intermediate susceptible; TMP/SMZ: trimethoprim/sulfamethoxazole; CLSI: Clinical Laboratory Standards Institute

The antibiogram of the antimicrobial agents that may warrant primary testing but may be reported only selectively (group B) for the *A. baumannii*, according to CLSI (2022), indicates that the highest resistance was recorded to FEP (75.6%), CRO (69.8%), and PTZ (66.3%). The highest levels of susceptibility were observed for colistin (58.1%), MIN (52.3%), and AK (51.2%). Intermediate susceptibility was demonstrated by 11.6% and 9.6% of the isolates to PTZ and TMP/SMZ, respectively. Doxycycline and cefiderocol were not included in the panel used for the antimicrobial testing, as demonstrated in Figure 2.

## Discussion

The percentage of MDRAB was highest in ICUs and neonatal units (48.8% and 30.2%, respectively) compared to other wards (21%). Within ICUs, the highest isolation was from the PICU (22.1%).

The majority of the *A. baumanni*i isolates were obtained from respiratory sites (55.8%). This finding is consistent with previous research conducted in Egypt (39.3%) [12] and Saudi Arabia (39.8%) [13]. Swabs were the next most frequent source of isolation (22.1%), surpassing the isolation rates reported in Egypt (14.3%) and Saudi Arabia (17.5%) [12,13]. The isolation rate of MDRAB from urine samples in our analysis (3.5%) was lower than those documented in Saudi Arabia [13] and Egypt [14] (5.8% and 32.5%, respectively). The prevalence of *A. baumannii* within ICUs and its recurrent isolation from ETTs can be attributed to the organism's capability to colonize medical devices through biofilm formation [15]. *A. baumannii *is frequently recognized as a colonizer of medical apparatuses in hospital environments. To ascertain whether these isolates resulted from colonization or true infection, it is imperative to evaluate the method of specimen collection and the clinical context of the patients involved.

This study demonstrated the antimicrobial susceptibility profile of MDRAB, resistance to beta-lactams, beta-lactam/beta-lactamase inhibitor combinations, carbapenems, aminoglycosides, fluoroquinolones, and MIN. In our findings, a remarkably elevated level of antibiotic resistance was observed across all antibiotic classes among the isolates. Resistance rates to third-generation cephalosporins in this article were found to be 83.7% for CAZ and 69.8% for CRO. Even higher rates of resistance have been reported in previous Libyan investigations, reaching up to 100% for both of the drugs [16-18]. Most doctors start with the third generation as an empirical treatment in most cases of severe infections. Current and previous study results show a high rate of resistance to these agents, and these practices need to be urgently reviewed. This requires a powerful and continuous surveillance and tracking system to detect changes in antibiotic resistance.

Furthermore, the resistance observed in our study is lower than that reported in Egypt (96.4%) and Saudi Arabia (100%) [12,13]. Additionally, the resistance to the fourth-generation cephalosporin FEP (75.6%) was found to be less than that reported in earlier studies in Libya [16-18], as well as in Egypt (97%) [12] and Saudi Arabia (100%) [13]. Resistance to beta-lactam/beta-lactamase inhibitors was detected in 90.7% of patients harboring MDRAB, aligning with previous findings in Libya [16], whereas resistance to piperacillin/tazobactam was noted at 66.3%. Higher resistance rates to piperacillin/tazobactam have been reported in Libya (100%) [17,18], and Egypt (96%) [14], whereas a low resistance (29.8%) was recorded in Iran [19].

In recent decades, the proliferating emergence of carbapenem-resistant *A. baumannii* isolates has constrained therapeutic alternatives. In our investigation, resistance rates to MEM and IMI reached 80.2% and 77.9%, respectively. Previous studies have certified a high prevalence of resistance to carbapenems too [16-18]. While AlAmri et al. reported lower resistance rates to MEM [13], Benmahmod et al. documented higher resistance rates [14]. Elevated levels of carbapenem resistance limit the therapeutic agents available for treating MDRAB infections in our healthcare facility.

The prevalence of resistance to aminoglycosides and TMP/SMZ was found to be lower in comparison to the resistance rates associated with beta-lactam antibiotics. A mere 48.8% exhibited resistance to AK. Abou Fayad et al. documented a resistance rate of 52% [17], whereas Zorgani et al. reported a significantly elevated rate of 81% [18]. Our findings indicated a resistance rate that was lower than those previously documented in Saudi Arabia, Egypt, and Tunisia [12,14,20]. Furthermore, a reduced rate of resistance was noted for TMP/SMZ at 46.5%. Two prior investigations conducted in Libya indicated resistance rates of 70% and 75% [16,17]. Elevated resistance figures of 77.9% and 92.5% were reported from Egypt [12,14]. Resistance to fluoroquinolones was high, reaching 81.4% for CIP; these rates have been documented previously in Libya [16-18].

The susceptibility of MDRAB to colistin in this investigation was determined to be 58.1%; however, as per the CLSI guidelines, the minimum inhibitory concentration (MIC) for colistin, as recorded by the automated system, is deemed unreliable, necessitating the use of colistin broth disk extrusion, broth microdilution, or colistin agar testing for accurate susceptibility assessment [11]; thus, reliance on the VITEK® 2 results for determining colistin susceptibility in *A. baumannii* is unwarranted. The release of the antibiotic susceptibility test report, including colistin, signifies a knowledge gap. Laboratory workers must receive education and training to keep up with the latest updates.

Minocycline and amikacin demonstrated the highest in vitro susceptibility against *A. baumannii* (52.3% and 51.2%, respectively), and as they can be administered as an IV formulation, they could be considered in empirical therapy. Minocycline IV is currently approved in the United States for use in Acinetobacter species infections, and recent studies indicate that minocycline remains highly active in vitro against *A. baumannii *[21].

Isolates showed a low level of intermediate resistance. Clinicians can increase the dose to improve the chance of treatment success, especially if infection is at the site where antibiotics usually concentrate [11].

The differences in resistance rates can be attributed to the differences in antibiotic prescribing practices and variations in adherence to infection control standards. In Libya, most clinicians start with third-generation cephalosporins as empirical treatment for severe infection despite the high resistance rate recorded by many studies. The specific guidelines for empirical therapy should be based on a local antibiogram within each healthcare setting.

This study had limitations. The retrospective design limits causal inference in resistance patterns, small sample numbers, and the data from a single center, so our result cannot be generalized to other healthcare settings. Inability to attribute the resistance to a specific mechanism due to lack of molecular data in addition to lack of clinical outcome data.

## Conclusions

In conclusion, serious infections associated with MDRAB are particularly challenging to treat because of the limited treatment options available, as the results confirmed the existence of high resistance rates among *A. baumannii* to both group A and B antimicrobial agents from different departments. The highest susceptibility recorded was to MIN and AK. Clinicians should consider using them in empirical therapy for severe infections associated with MDRAB.
